# Supplementary material for: Ultrafast multi-cycle terahertz measurements of the electrical conductivity in strongly excited solids
Source: Nat Commun. 2021 Mar 12;12:1638. doi: 10.1038/s41467-021-21756-6 (PMC7977037; doi:10.1038/s41467-021-21756-6)
Supplement: Supplementary file 1 — Supplementary Information [file 41467_2021_21756_MOESM1_ESM.pdf]

Supplementary Information for

Ultrafast Multi-cycle Terahertz Measurements of the Electrical Conductivity in Strongly Excited Solids

Z. Chen[1*], C.B. Curry[1,2], R. Zhang[2], F. Treffert[1,3], N. Stojanovic[4,5], S. Toleikis[4], R. Pan[4], M. Gauthier[1], E. Zapolnova[4], L.E. Seipp[1,5], A. Weinmann[1,6], M.Z. Mo[1], J.B. Kim[1], B.B.L. Witte[1,7], S. Bajt[4,8], S. Usenko[4,9,10], R. Soufli[11], T. Pardini[11], S. Hau-Riege[11], C. Burcklen[11], J. Schein[6], R. Redmer[7], Y.Y. Tsui[2], B.K. Ofori-Okai[1], and S.H. Glenzer[1*]

[1]SLAC National Accelerator Laboratory, Menlo Park, California 94025, USA

[2]University of Alberta, Edmonton, Alberta T6G-1H9, Canada

[3]Technische Universität Darmstadt, 64289 Darmstadt, Germany

[4]Deutsches Elektronen-Synchrotron DESY, 22607 Hamburg, Germany

[5]DLR–Institute for Optical Sensor Systems, Rutherfordstraße 2, 12489 Berlin, Germany

[6]Universität der Bundeswehr München, 85579 Neubiberg, Germany

[7]Institut für Physik, Universität Rostock, 18059 Rostock, Germany

[8]The Hamburg Centre for Ultrafast Imaging, Hamburg, Germany

[9]Institut für Experimentalphysik, Universität Hamburg, 22671 Hamburg, Germany

[10]European XFEL GmbH, 22869 Schenefeld, Germany

[11]Lawrence Livermore National Laboratory, Livermore, 94550 California, USA

*To whom correspondence should be addressed; E-mail: zchen@slac.stanford.edu, glenzer@slac.stanford.edu

Supplementary Text

XUV-FEL pulse energy deposition and temperature estimation

The 1/e attenuation length of 13.6 nm (photon energy = 91.2 eV) XUV-FEL in gold is 20 nm (*1*), which results in about 77% absorption of the incident XUV-FEL pulses. The energy absorption is dominated by the ionization of the $5d$ and $5p$ electrons (*2*), and the absorption ratio of XUV energy is expected to be almost constant in our experimental condition. Taking into account the efficiency of the XUV optics, at maximum pulse energy of 160 μJ, about 75 μJ is focused on the sample surface, resulting in a maximum intensity of 5×10^{11} W/cm^2. The maximum absorbed energy density is 1 MJ/kg, corresponding to a photon ionization rate of 0.02 eV using the 91.2 eV XUV photons. The ionized electrons are expected to be thermalized in 100 fs time scale (*3*), leading to a maximum electron temperature of 16,800 K, or 1.5 eV according to the two-temperature model calculations. Because this temperature is only a fraction of the Fermi energy (5.5 eV in gold), the electrons are expected to follow the Fermi distribution. It is also worth to mention that XUV-FEL pulses generated from the self-amplified spontaneous emission (SASE) usually contain multiple random spikes whose peak intensities can be several times higher than the average intensity (*4*). In our experiment, they are not expected to cause any change to the energy absorption. This is consistent with previous measurements of aluminum (*5*) that have shown linear absorption for intensities up to 10^{14} W/cm^2.

Earlier studies in 400 nm ultrafast laser excited Au foils have shown that the longitudinal thermal gradient in 30 nm gold thin foils is eliminated within 500 fs after laser excitation. Such observation is ascribed to the ballistic electron energy transport (*6, 7*). In this study of XUV-FEL excited 30 nm-thick gold, we find that our samples are also uniformly heated within 500 fs. Here, electron thermal diffusion eliminates the thermal gradient. To quantify this process, we perform the two temperature model (TTM) calculations (*8*),

$$C_{\mathrm{e}}(T_{\mathrm{e}}(z,t))\frac{\partial T_{\mathrm{e}}(z,t)}{\partial t} = \frac{\partial}{\partial z}(\kappa_{\mathrm{e}}\frac{\partial T_{\mathrm{e}}(z,t)}{\partial z}) - g_{\mathrm{ei}}(T_{\mathrm{e}}(z,t) - T_{\mathrm{i}}(z,t)) + S(z,t) \qquad \text{(1a)}$$

$$C_{\mathrm{i}}(T_{\mathrm{i}}(z,t))\frac{\partial T_{\mathrm{i}}(z,t)}{\partial t} = g_{\mathrm{ei}}(T_{\mathrm{e}}(z,t) - T_{\mathrm{i}}(z,t)) \qquad \text{(1b)}$$

where C_{e} and C_{i} are the specific heat capacities of electrons (*9*) and ions (*10*), κ_{e} is the electron thermal conductivity (*11*), g_{ei} is the electron-ion energy coupling parameter determined from recent experimental studies (*12*), S is the XUV-FEL pulse energy deposition rate, T_{e} and T_{i} are the time-dependent electron and ion temperatures along the longitudinal direction z. Within 500 fs after the XUV-FEL heating pulse, 98% of the absorbed energy stays in the electronic system. Therefore, we calculate the time-dependent behavior of T_{e} along the longitudinal direction z. Figure S1 shows an example of a sample heated by a 150 fs FWHM XUV-FEL pulse with absorbed fluence of 52mJ/cm^2, corresponding to an averaged absorbed energy density of ΔE=0.9 MJ/kg over the sample thickness, These results indicate that electron thermal diffusion eliminates the thermal gradient inside the sample. $\Delta\varepsilon(z) = \int_{-\infty}^{\infty} S(z,t)dt$ is the local absorbed

energy density along z direction, which is plotted in Fig. S1(a). Fig. S1(b) shows the corresponding T_e profiles at different time delays. Here t=0 fs is the time when the XUV-FEL pulse reaches peak intensity. We find that although $\Delta\varepsilon$ from XUV-FEL energy deposition attenuates from $\sim$1.8 MJ/kg at the front surface to $\sim$0.4 MJ/kg at the rear surface, at t= 500 fs no temperature gradient is found owing to electron thermal diffusion. Therefore, the thermal diffusion term in Eq. 1(a) is not included in subsequent calculations beyond 500 fs and the sample is considered to be uniformly heated. Because both XUV-FEL (this work) and optical-laser (*13*) can uniformly excite the 30nm gold films within 500fs, it also justifies our comparison of the THz conductivity measured from this work with previously reported optical conductivity data (*13*).

The XUV-FEL focal spot on target was measured to be 225 $\mu m \times$ 255 μm FWHM, cf. Fig. S2(a, b). Fairly uniform T_e distribution is established in lateral directions after the XUV-FEL pulse. An example of the temperature profile for an averaged absorbed energy density of 0.9 MJ/kg at t=300 fs is shown in Fig. S2(c, d), indicating uniformity of 12$\%$ RMS within the 250 μm diameter THz probe region. Using TTM calculations with a T_e-dependent electron thermal conductivity (*11*), we find that thermal diffusion in the lateral direction is negligible within the 30 ps time window of our measurement, and the lateral profile of the absorbed energy density is expected to stay unchanged.

The averaged energy density is estimated by dividing the absorbed XUV-FEL pulse energy by the mass of the sample within the THz probed area of 250 μm diameter. The electrical conductivity is then measured at various absorbed energy densities and peak electron temperatures using calibrated Zr thin film filters to precisely attenuate the XUV-FEL pulse energies. (*14*).

Using Eqs. 1, the temperatures for different excitation conditions are computed. Fig. S3(a) shows both the electron and ion temperatures at 0.7 ps after XUV-FEL excitation as a function of absorbed energy density. The temporal dependent temperatures for excitation energy density of 0.89MJ/kg are shown in Fig. S3(b). In the TTM calculations, we also include the 20$\%$ error-bar from the electron-ion energy coupling factor g_{ei} reported in (*12*), and the resulting uncertainties are shown in the pink and light-blue area. The TTM calculations in our experimental conditions show electron-ion equilibrium time scales well in excess of 15ps, which is significantly longer than the experimental observations at much lower excitation energy density conditions (*15, 16*). This behavior is in agreement with expectations because the electron-ion equilibrium time scale increases with the absorbed laser energy density (*15*).

Multi-cycle THz data analysis

The THz signal is measured by the single-shot electro-optics sampling method with the implementation of balanced detection (*17*). Fig. S4 shows examples of the balanced detection that correspond to the data presented in Fig. 2 of the main text. We can see that subtracting the signal recorded by one polarization state of the sampling laser (left signal) by another one recorded by the opposite polarization state (right signal) results in significant improvements to the signal to noise ratio of the data.

The electrical conductivity σ measured by the multi-cycle THz radiation is analyzed in the time domain using the following equation (*18*),

$$|\sigma| = \frac{2}{Z_0 d}(\frac{1}{|t_r|} - 1) \tag{2}$$

where Z_0 =377 Ω is the free space impedance, d=30 nm is the sample thickness, $|t_r| = \frac{|E_\text{i}|}{|E_\text{i}|}$ is the amplitude of the electric field that transmits through the sample. $|E_\text{i}|$ and $|E_\text{i}|$ are integrated for each THz cycle to determine $|t_r|$. The boundaries of the cycles are defined by the measured $|E_\text{i}|$ when $\frac{\Delta I}{I_0}$ passes 0 on the falling edges. $|\sigma|$ of each unheated sample is obtained by averaging the measurements from all 11 cycles, and $|\sigma|$ from the heated sample is obtained from each individual THz cycle. Since $\frac{\Delta I}{I_0}$ increases by about an order of magnitude from cold solid to hot liquid, we achieve excellent signal-to-noise ratio (SNR) for each single cycle.

The amplitude of the THz radiation shows only 4% RMS fluctuations from shot to shot, cf. Fig. S5, which is negligible compared to the large increase in $\frac{\Delta I}{I_0}$. Consequently, we are able to use $|E_\text{i}|$ and $|E_\text{i}|$ measured on subsequent shot pairs to obtain $|t_r|$ and σ. The temporal jitter between the THz-FEL and the ultrafast laser for electro-optic(EO) sampling is of order 50 fs RMS as shown in Fig. S5 (a) and (b). To average the pre-shot data taken on the same sample, the shot-to-shot jitter is corrected by an auto-correction function. Fig. S5 (c) and (d) show the corrected results of Fig. S5 (a) and (b). The same method is used to temporally overlay the pre-shot, on-shot and post-shot data in order to determine the individual electrical conductivity for each cycle. The small amount of phase retardation (<30 fs) caused by the thin film samples is not resolved. Our analysis utilizes the absolute values of the transmission.

The frequency components in each terahertz cycle is shown in Fig. S6. The peak frequencies of the first 9 cycles are within 2-3 THz, and the last two cycles peak at lower frequencies because they contain contribution from single-bend emission that is generated when the electrons exist the THz undulator. The full width half maximum bandwidth of each single-cycle is in the range of 3-4 THz.

Fig. S7 shows the comparison of the time resolved electrical conductivity determined from our analysis with the more commonly applied THz time-domain spectroscopy (THz-TDS) analysis. The latter uses single-cycle THz pulse that is generated when the electron bunches are deflected by the dumping magnet located after the undulators (*19, 20*) without operating the THz undulator. The temporal and spectral profiles of the single-bend-emitted THz radiation are shown in the inset of Fig. S7. Here the conductivity is determined using the same Eq. 2 but solved in the frequency domain. The frequency-dependent E-field amplitude of the THz radiation is obtained by Fourier transformation of the time domain data. An example for a single-shot measurement carried out at 5$\pm$1 ps after XUV-FEL excitation is shown in Fig. S8. From Fig. S8 (c), the values of $|\sigma|$ within 1-3 THz are frequency independent and they are averaged to obtain the conductivity data. Both data sets obtained from time- and frequency-domain analysis are in agreement, corroborating the robustness of our multi-cycle THz analysis. In addition, the multi-cycle THz radiation measures the evolution of the electrical conductivity over a larger time window with better temporal resolution. It allows the time-resolved measurements of samples that have varying initial conditions, such as defected or radiation damaged samples (*21*).

Comparing the Drude model and the Drude-Smith model fit to the Broadband conductivity data

To confirm that the Drude model is sufficient to describe the frequency dependent electrical conductivity of our free-standing nano-foil samples, we also use the Drude-Smith model to fit the broadband conductivity data. The Drude-Smith model has similar format as the Drude model:

$$\sigma(\omega) = \frac{n_\mathrm{e} e^2 \tau}{m(1 - i\omega\tau)} \left[1 + \frac{C}{1 - i\omega\tau}\right] \tag{3}$$

where n_e is the carrier electron density, e is the electron charge, m is the electron effective mass, τ = 1/ν_e is the electron scattering time, which is the inverse of the total electron momentum scattering frequency ν_e, and the parameter C, ranging between 0 and -1, is introduced to account for electron back scattering from nano-structures in the sample (*22, 23*). It returns to the Drude model when C=0. We take the real part of Eq. 3 to fit the broadband conductivity data, i.e. $\sigma_r(\omega)=\frac{n_\mathrm{e} e^2 \tau}{m(1+\omega^2\tau^2)}\left[1 + \frac{C(1-\omega^2\tau^2)}{(1+\omega^2\tau^2)}\right]$. Here, we first determine C at room temperature with $n_\mathrm{e} = 5.9 \times 10^{28} m^{-3}$, corresponding to one carrier electron per atom (*24*), and the electron effective mass $m = 9.1 \times 10^{-31}$kg in gold that is equal to its rest mass (*25*). Including the THz data, the optical data at 1.55 eV, and the four point probe data, we calculate C=-0.17$\pm$0.16 for our thin foil samples, where the error bar is determined from the fit through the boundaries of the of the experimental error. Better determination of the C parameter requires more careful measurements of the DC, THz and optical continuities on the same samples, or even includes additional measurements in the mid-infrared regime. We find that the frequency response of $\sigma_r(\omega)$ is effectively constant for photon energies less than 0.012 eV, resulting in $\sigma_r \approx \sigma_0$ in this regime. In Fig. S9, the solid and dotted curves show the frequency-dependent conductivity values fit by the broadband Drude and Drude-Smith models respectively. These two curves are almost identical, indicating that the electron back scattering does not play a significant role in our samples. This is in agreement with previous studies (*22*) that have found that the Drude-Smith model only show significant difference from the Drude model when gold foil thickness is below 10 nm. Therefore, the broadband electrical conductivity is adequately described by the Drude model.

Electron scattering frequencies and electron densities deduced from the Drude and Drude-Smith models

We apply both models (*23, 26*) to study the relationship between our measured electrical conductivity data at THz frequencies and the real part of the optical conductivity σ_r measured at a photon energy of 1.55 eV (*13*). The corresponding broadband electrical conductivity data are shown as a function of photon energy in Fig. 3 of the main text. These data are measured shortly after excitation when T_e is near its maximum but T_i remains low. In Fig. S10 we show the electron scattering frequencies (ν_e, ν_ee and ν_ei) similar to Fig. 5 in the main text, except that the data are determined by the Drude-Smith model fit. These data are about 15% lower compared to the Drude model. This value is within the uncertainty of the data and does not affect the conclusions of this study.

In Fig. S11(a), the electron densities n_e are determined from the fits of both models and are found to be almost identical. They increase by $\sim 30\%$ when the T_e increases from 300 K to 16000 K, which is much smaller than the change in electron scattering frequencies. In Fig. S11(b), we plot ν_ee as functions of T_e^2, which shows that ν_ee follows the Fermi-liquid scaling (*24*) even when T_e reaches 1/4 of Fermi temperature.

Relation between THz conductivity and DC Electrical conductivity

We estimate the absolute value of the complex electrical conductivity using Eq. 2, which is equivalent to Eq. 1 in the main text. This comes from the Tinkham equation, which is an approximation for conducting samples in the long wavelength limit (wavelength of the electric field is much larger than the sample thickness) (*18, 22*).

Here, we evaluate the accuracy of the Tinkham equation and the approximations of $\sigma(\omega) \approx \sigma_r(\omega) \approx |\sigma(\omega)| \approx \sigma_0$ in the THz regime. We first use the Drude-Smith model to construct the complex electrical conductivity σ as a function of electron scattering frequency ν_e. The corresponding THz field transmission t_r through a 30 nm foil is calculated exactly using the characteristic matrices method (*27*). Substituting $|t_r|$ in Eq. 2, the absolute value of the conductivity is determined, which is called $|\sigma_{Tk}|$ as the result of the Tinkham equation. The ratio of $|\sigma_{Tk}|$ over our constructed $|\sigma|$ as functions of ν_e/ω is presented in Fig. S12 (a), where $\sim 1\%$ over estimation is found for ν_e at room temperature conditions, and almost exact solutions are found at higher ν_e for excited solids at high T_e conditions. We also plot $|\sigma|/\sigma_0$, $\sigma_r/|\sigma|$ and σ_r/σ_0 in Fig. S12 (b), where σ_r and σ_0 are obtained for the same Drude-Smith model calculations. Obviously, the approximations of $\sigma \approx \sigma_r \approx |\sigma| \approx \sigma_0$ are accurate, i.e. less than 3% deviation is found at room temperature, and no deviations for heated conditions.

Electron-electron and Electron-ion scattering frequency obtained from the THz conductivity

ν_e is the sum of electron-electron and electron-ion scattering frequencies ν_ee and ν_ei (*11, 24*), which are primarily dominated by T_e and T_i respectively, i.e. $\nu_\mathrm{e}(T_\mathrm{e}, T_\mathrm{i}) \approx \nu_\mathrm{ee}(T_\mathrm{e}) + \nu_\mathrm{ei}(T_\mathrm{i})$. At room temperature, $\nu_\mathrm{ei} \gg \nu_\mathrm{ee}$, thus $\nu_e \approx \nu_\mathrm{ei}$ (*24*).

The T_e-dependent electron scattering frequency, ν_ee, is derived from ν_e measured at fixed time delay (t=0.7±0.2 ps) shortly after photo-excitation with different absorbed energy densities, $cf.$ Fig. S11(a). At these delays T_e is near its maximum but T_i changes by much smaller amount as shown in Fig. S3(a). The scattering frequency is thus determined by $\nu_\mathrm{ee}(T_\mathrm{e}) = \nu_\mathrm{e} - \nu_\mathrm{ei}(T_\mathrm{i})$, where T_e and T_i are determined from TTM calculations and ν_ei is estimated based on its linear dependence of T_i , i.e. $\nu_\mathrm{ei}(T_\mathrm{i})/\nu_\mathrm{ei}(300K)$=$T_\mathrm{i}$/300K (*24*).The resulting values for $\nu_\mathrm{ee}(T_\mathrm{e})$ are shown in Fig. 4(c) of the main text. In Fig. S11 (b), we also show the linear scaling of ν_ee as a function of T_e^2.

Extrapolating the DC conductivity from measured optical conductivity and comparison with the reported experimental data

In Fig. 3 of the main text, we show the extrapolated of the 800 nm optical conductivity reported in (*13*) to the low frequency regime based on the Drude model, where both the real and

imaginary parts of the conductivity are determined from the simultaneous measurement of the reflection and transmission. The original Drude model is applied in the extrapolation,

$$\sigma(\omega) = \sigma_r(\omega) + \sigma_\mathrm{i}(\omega) = \frac{\sigma_0}{1 - i\omega\tau} \tag{4}$$

where σ_r and σ_i are the real and imaginary parts of the optical conductivity, ω is the angular frequency of the 800 nm light, and τ is the electron scattering time. Then the DC conductivity σ_0 can be calculated using σ_r and σ_i by re-arranging the above equation,

$$\sigma_0 = \sigma_r(\omega)[1 + \frac{\sigma_\mathrm{i}^2(\omega)}{\sigma_r^2(\omega)}] \tag{5}$$

Fig. S13 shows the comparison of the DC conductivity values determined from our THz measurement, i.e. $\sigma_0 \approx |\sigma|$, with the data extrapolated from the optical conductivity measurements in (*13*). The latter is about a factor of 2 lower than the measured results. For comparison, we also include other DC conductivity of isochorically heated gold extrapolated from optical conductivity data measured with 800nm lasers (*28–30*). In (*28*), a modified Drude model accounting for the non-Drude components in the imaginary part of the optical conductivity is applied to extrapolate DC conductivity from the optical conductivity reported in (*13*). In (*29*), the optical conductivity at 2 - 3ps after laser heating is used, where higher ionic temperature than this study is expected, and the authors use Eq. 5 to extrapolate DC conductivity. In (*30*), only the electron density n_e and the electron scattering frequency ν_e are reported, and we use them to construct the DC conductivity data following the original Drude formula. We can see that all these extrapolated DC conductivity data are lower than our results measured by the THz-FEL, consistent with the extrapolations shown in Figure 3 of our main text.

Determine XUV-THz overlap in time using silicon sample

To find the temporal overlap between the XUV-FEL and THz-FEL pulses, we use a 500μm-thick high resistivity single-crystal silicon wafer (2.4$\times 10^3 \Omega$.m) as a timing tool. We tune the XUV pulses energy to be lower than the damage threshold of the silicon wafer so that the measurement can run continuously without replenishing the sample. Then we scan the physical path length of the THz-FEL pulse relavent to the XUV-FEL pulse, and also adjust the time delay of the EO-sampling optical pulses accordingly. When the XUV pulses arrive on the sample later than the THz pulses, we observe no change of THz transmission through the sample comparing the measurements with and without XUV excitation, and when the XUV pulses arrive earlier, the photo-excited and thermal-excited free carriers causes strong shielding of the THz pulse and significantly reduce its transmission. This phenomenon agrees with the studies of optical laser excitation of silicon (*31*). We continue the scan until part of the THz-FEL cycles arrives earlier than the XUV-FEL as shown in Fig. S14. Once the temporal overlap is found, we switch to the free-standing gold thin foils to start our experiments.

Electron-ion scattering frequencies obtained from experimental measured ion-ion structure factors

The Ziman formulation and its extended versions (*32, 33*) determine the electrical conductivity of disordered and liquid metals. It estimates the electron-ion momentum scattering frequency ν_{ei} from the ion-ion structure factor (S_{ii}), which is usually obtained from theoretical calculations (*34, 35*). Recent ultrafast electron diffraction (UED) studies (*12*) allowed us to experimentally determine S_{ii}. Here we use these data to calculate ν_{ei} using the following formula (*36*):

$$\nu_{\text{ei}} = \frac{n_{\text{i}} m_{\text{e}}}{2p_{\text{F}}^3} \int_0^{2p_{\text{F}}/\hbar} \frac{dq}{2\pi} \left| \frac{4\pi e^2}{q^2 + k_{\text{e}}^2} \right| q^3 S_{\text{ii}}(q) \tag{6}$$

where n_{i}=5.9×10^{28}/m^{-3} is the ionic density, m_{e} is the electron mass, p_{F} is the Fermi momentum, e is the of electron charge, k_{e} is the electronic screening vector obtained from Ref. 37.

In the UED experiments (*12*) performed at approximately the same energy densities as this study, we obtain the structure factor given by,

$$S_{\text{ii}}(q) = \frac{I(q)}{N(\gamma f_{\text{e}})^2} \tag{7}$$

where $I(q)$ is the elastic scattering intensity obtained from the diffraction data (*21*), N is a calibration factor for converting the measured intensity units into an absolute scale, γ is the Lorentz factor to account for relativistic effects of the 3.2 MeV electrons, and f_{e} is the electron scattering form factor for gold (*38*). The $S_{\text{ii}}(q)$ data for several T_{i} conditions at the absorbed energy density of 0.68 MJ/kg are shown in Fig. S15. The instrumental resolution of 0.26$\AA^{-1}$ FWHM is deconvoluted from the measured structure factor, *cf.* Fig. S15. The upper bound of the integration in Eq. 6 is marked in Fig. S15. The broadening of the solid peak and the rise of liquid peak results into and increase of ν_{ei}, in agreement with the THz data. At significantly high T_{i} conditions beyond the superheating limit, the crystalline structure transits into liquid rapidly (*12, 39*), and the Drude-Smith model description of grain boundary back scattering may not hold any more. Therefore, in Fig. 4(d) of the main text, we only focus on the comparison of ν_{ei} up to 1.3 times of the nominal melting temperature before significant melting occurs (*12, 39*).

Supplementary Figures

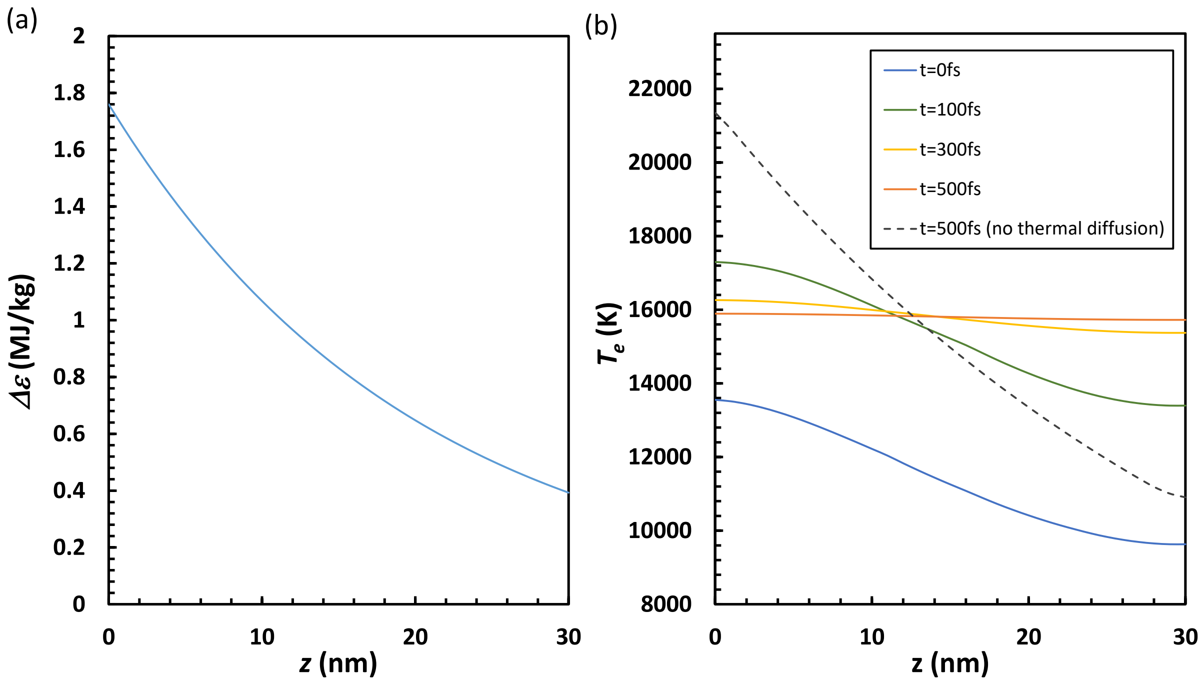

Figure S1: (a) The local absorbed energy density $\Delta\varepsilon$ inside a 30 nm gold thin film as a function of distance from the front surface (z). Here the sample is heated by a 150 fs, 13.6 nm FEL pulse, resulting in an absorbed energy density of 0.9 MJ/kg. (b) shows the electron temperature (T_{e}) as a function of z at time delays of t = 0 fs, 100 fs, 300 fs and 500 fs.

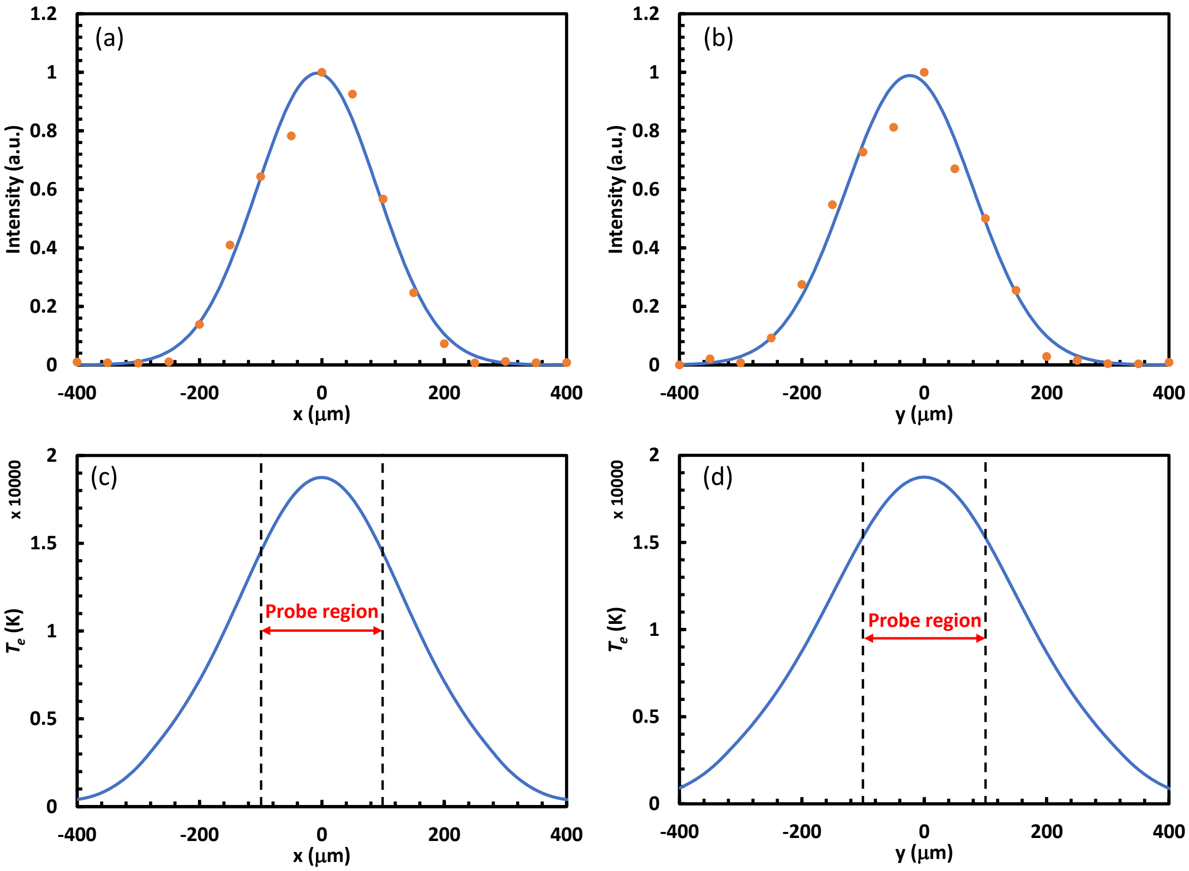

Figure S2: (a) and (b) show both the measured XUV-FEL focal spot (orange data points) and Gaussian profile fit (blue lines) along x (horizontal) and y (vertical) axes respectively. The intensities are in arbitrary unit (a.u.) that are normalized to the peak intensity. (c) and (d) show the calculated T_e profile along x and y axes for an average energy density of 0.9 MJ/kg within the THz probed area at t=300fs. The boundaries of the probe area are marked by vertical dashed lines.

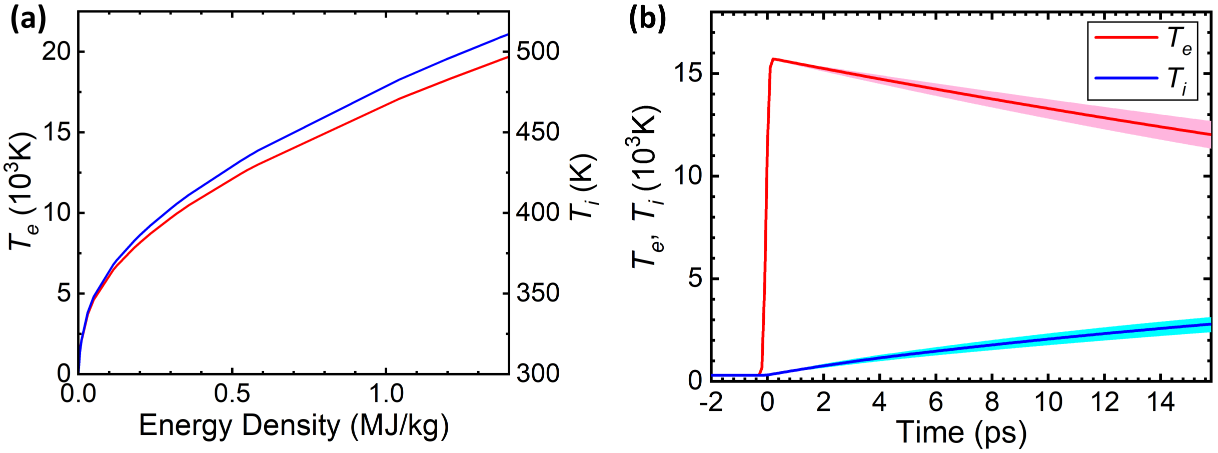

Figure S3: Two temperature model calculated (a) electron temperature T_e and ion temperature T_i as functions of absorbed energy density at t=0.7ps after the XUV-FEL excitation, and (b) temporal evolution of T_e and T_i at an absorbed energy density of 0.89 MJ/kg. The pink and the light blue shadows represent the error estimation of the TTM calculations

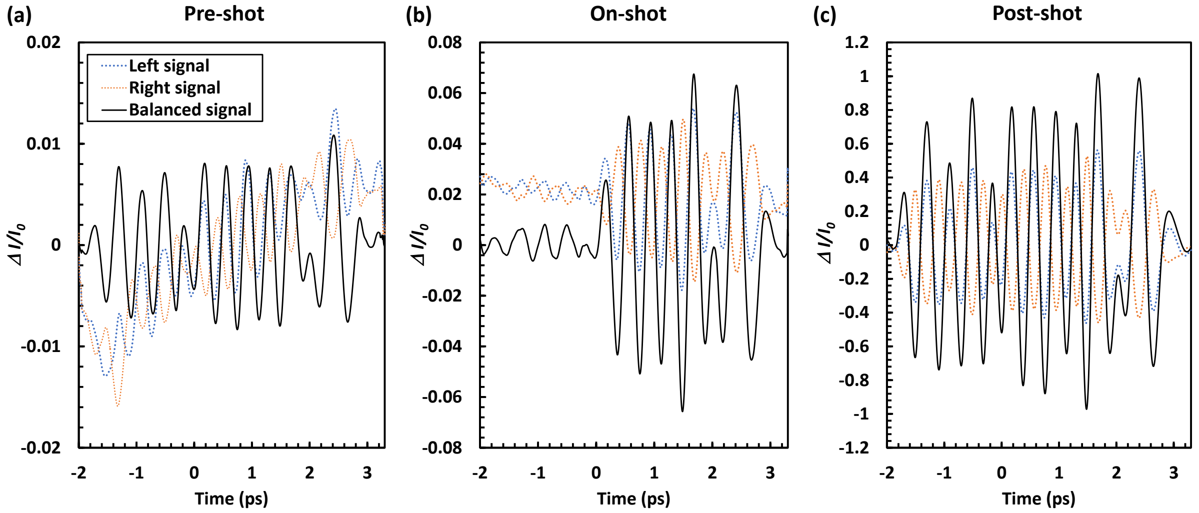

Figure S4: Example data of the THz electric field acquired by the balanced detection for (a) pre-shot, (b) on-shot and (c) post-shot measurements that correspond to the data shown in Fig. 2 of the main text. The XUV-FEL heating pulse arrives at 0 ps during the on-shot measurement. No XUV-FEL pulse is applied during the pre-shot and post-shot measurements.

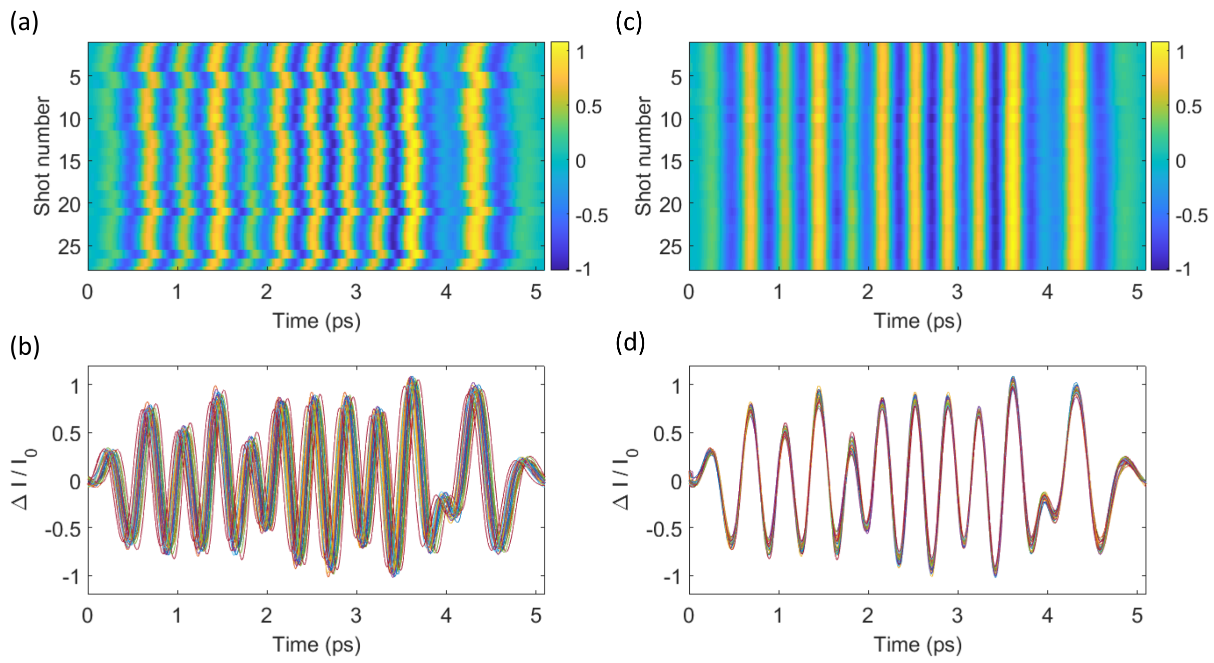

Figure S5: Sequence of 28 multi-cycle THz pulses that were obtained every 5 seconds. (a) shows the original time dependent profile without jitter correction, and (b) is the result after jitter correction. (c) and (d) overlay the individual lineout plots from (a) and (b) respectively.

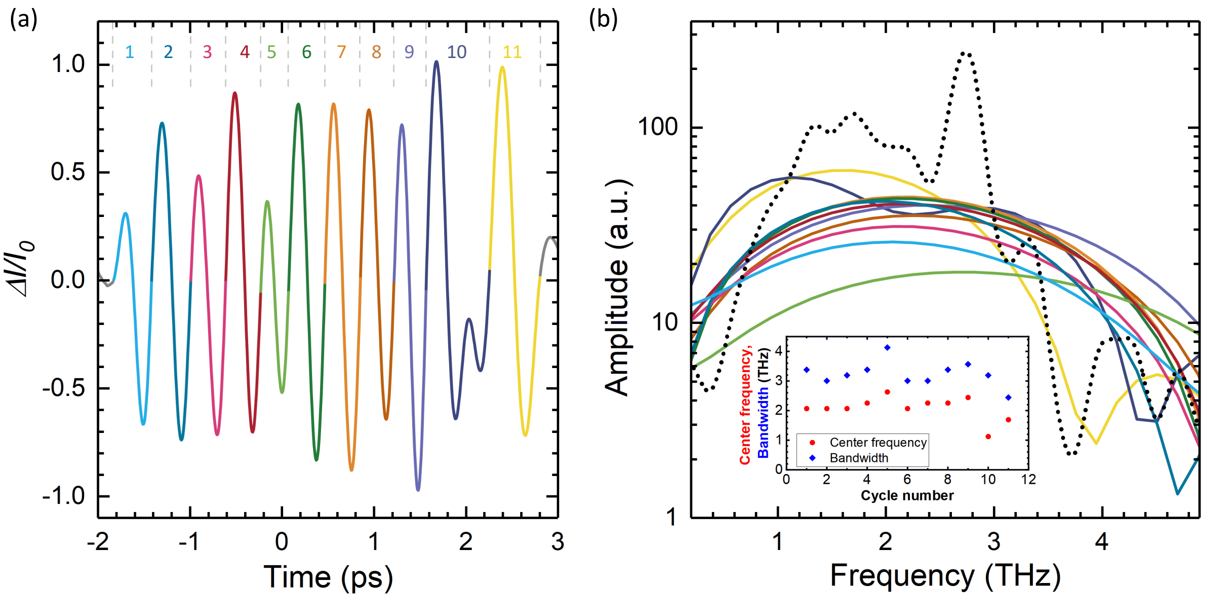

Figure S6: Frequency components of the individual terahertz cycles. (a) shows the single-shot measured THz cycles used in our time-resolved conductivity measurements, where each cycle is shown with a different color. (b) uses the same color code to show the amplitudes of the corresponding frequency components, and the dashed line represents the sum of the cycles. The inset in (b) shows the peak frequency and bandwidth of each THz cycle (the cycle numbers follow the ones shown in (a)).).

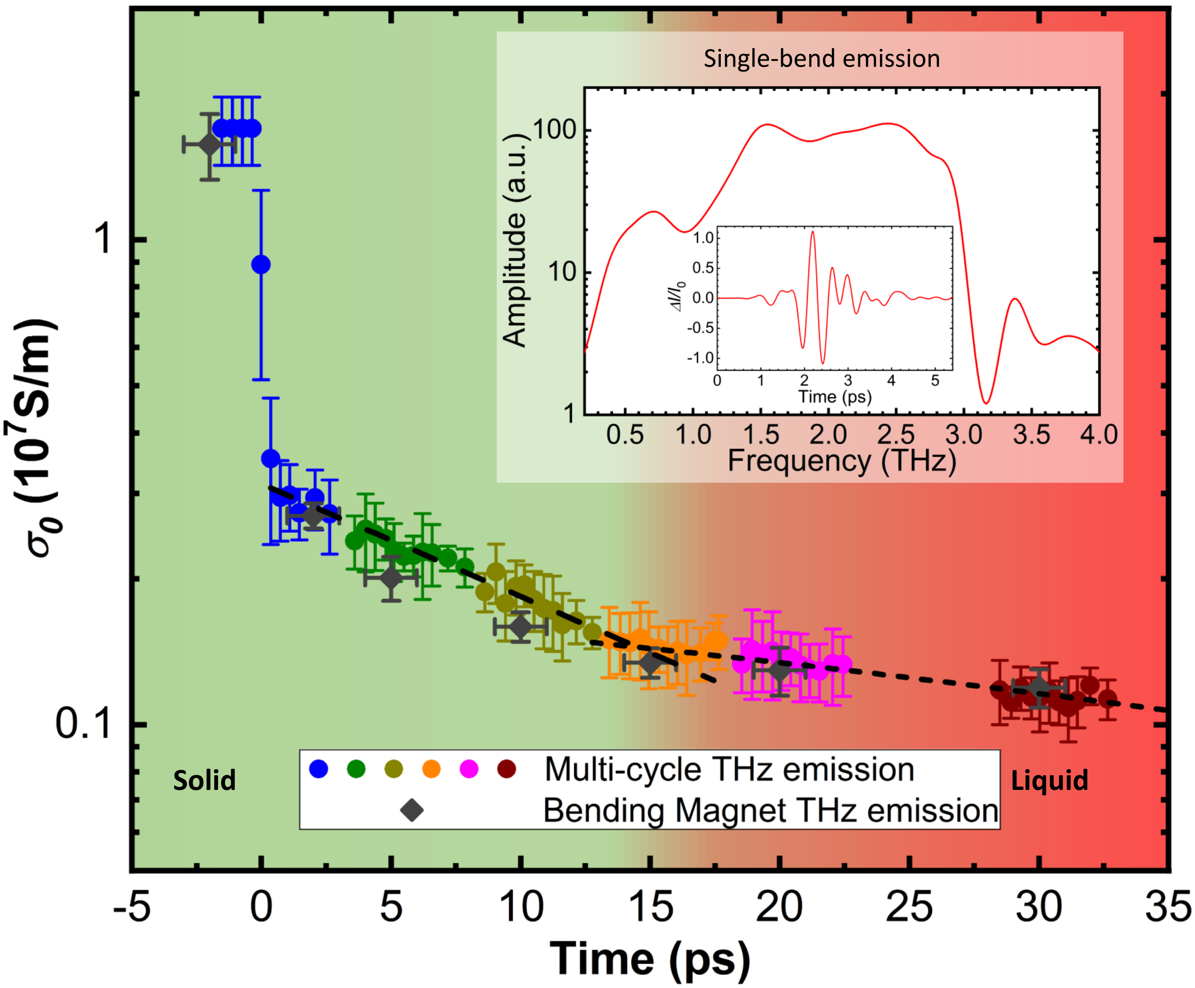

Figure S7: Time-resolved electrical conductivity σ_0 of gold (logarithmic vertical axis) at mean excitation energy density of 0.89±0.18 MJ/kg measured by multi-cycle and single-bend-emitted THz pulses. The data are measured with multi-cycle (circles of multiple colors) and single-bend-emitted THz (grey diamonds) pulses. Each section of multi-cycle measurement is averaged over 4-6 shots, and each data point from the single-bend-emitted measurement is averaged over 6-8 shots. The vertical error-bars include the standard deviation and the systematic error of the measurements, and the horizontal error bars in the single-bend-emitted THz measurements represents the width of the pulses. The multi-cycle THz measured σ decay after 0 ps is fitted by a two-segment exponential decay function represented by dashed and dotted lines respectively, and the intersection of the two segments is found at t=14.5 ps determined by the minimum sum of squared error. The inset shows spectral and temporal profiles of the single-bend-emitted THz pulses.

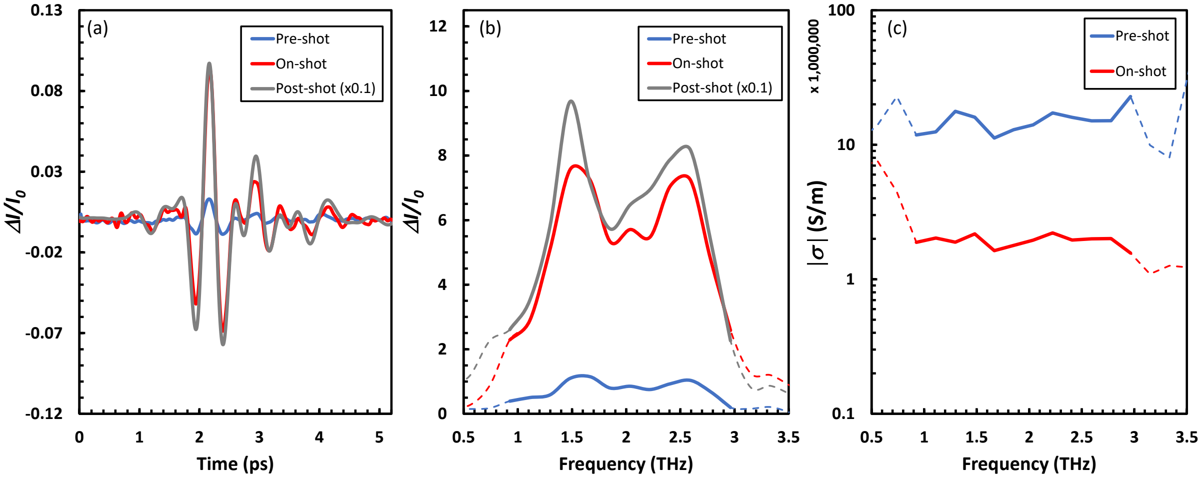

Figure S8: Single-shot measurement of σ at 5±1 ps after XUV-FEL exciation for absorbed energy density of 0.93±0.18 MJ/kg. (a) shows the measured E-field amplitude for pre-shot, on-shot and post-shot conditions, and (b) shows the corresponding frequency dependent amplitude obtained from Fourier transform. The obtained frequency dependent $|\sigma|$ is given in (c).

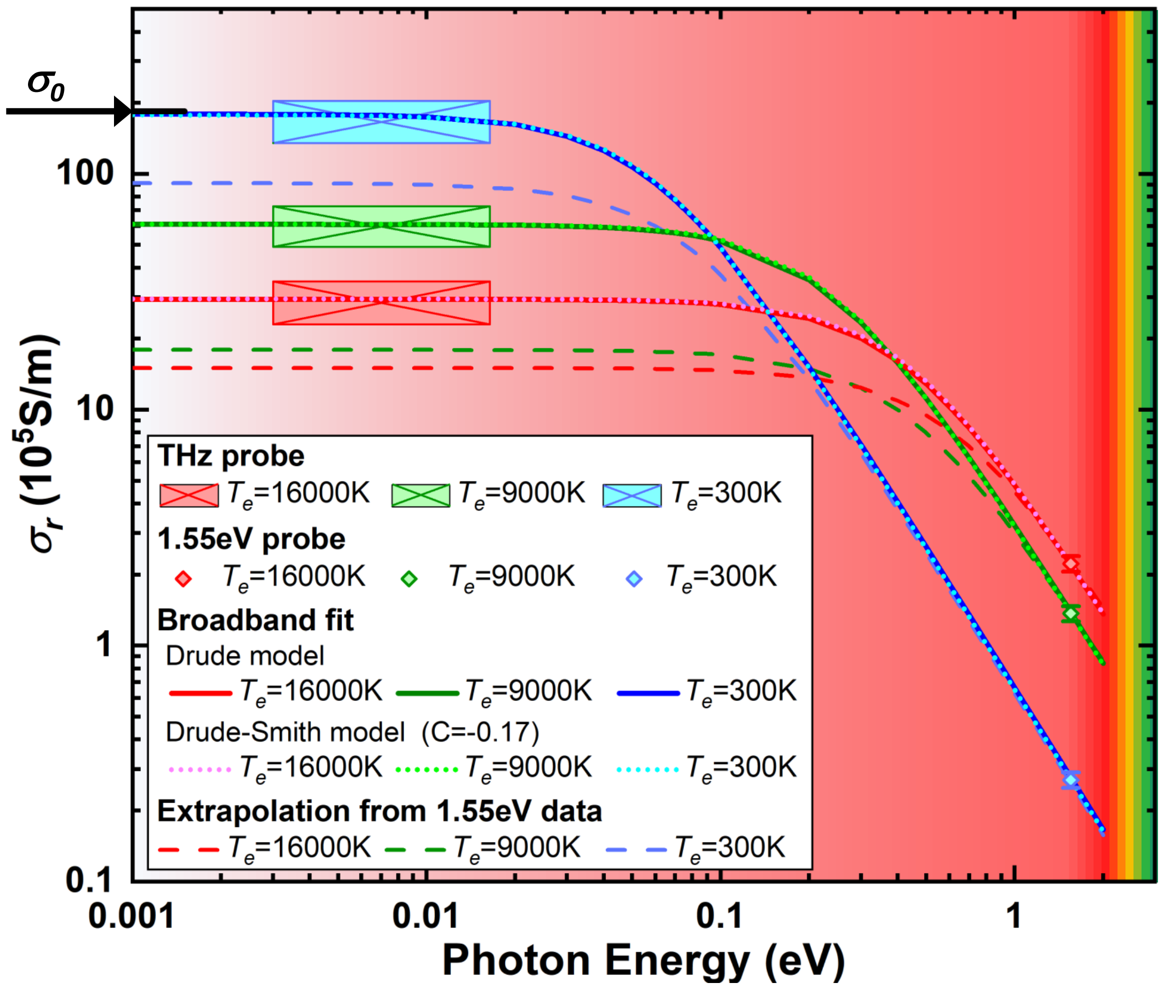

Figure S9: Comparison of the Drude model and Drude-Smith model fit to the electrical conductivity as a function of photon energy for various excitation conditions of solid gold. The broadband electrical conductivity (real part, σ_r) at electron temperatures of T_e =300 K (blue), 9000±900 K (green) and 16000±1500 K (red) are indicated by different colors; the THz conductivity at small photon energies (0.75-3.75 THz in frequency, or 3-15.5 meV in photon energy) are shown as square blocks that cover the range of frequency components in the individual THz cycles that are used for the measurements (horizontal dimension) and precision of the inferred conductivity (vertical dimension), the optical conductivity at higher photon energy (1.55 eV) from (*13*) are presented as diamonds, and the DC conductivity at room temperature measured with four-point probe is pointed out by a black arrow. The dotted curves represent the Drude-Smith model (*23*) fit through the high and low frequency data with a best fit back scattering parameter C=-0.17, which overlap with the Drude model fit represented by solid curves, and the dashed curves are extrapolated from the optical conductivity at 1.55 eV using the Drude model. The conductivity measured with THz radiation agrees well with the DC conductivity from four-point probe measurements, while the extrapolation from optical measurements shows significant discrepancies.

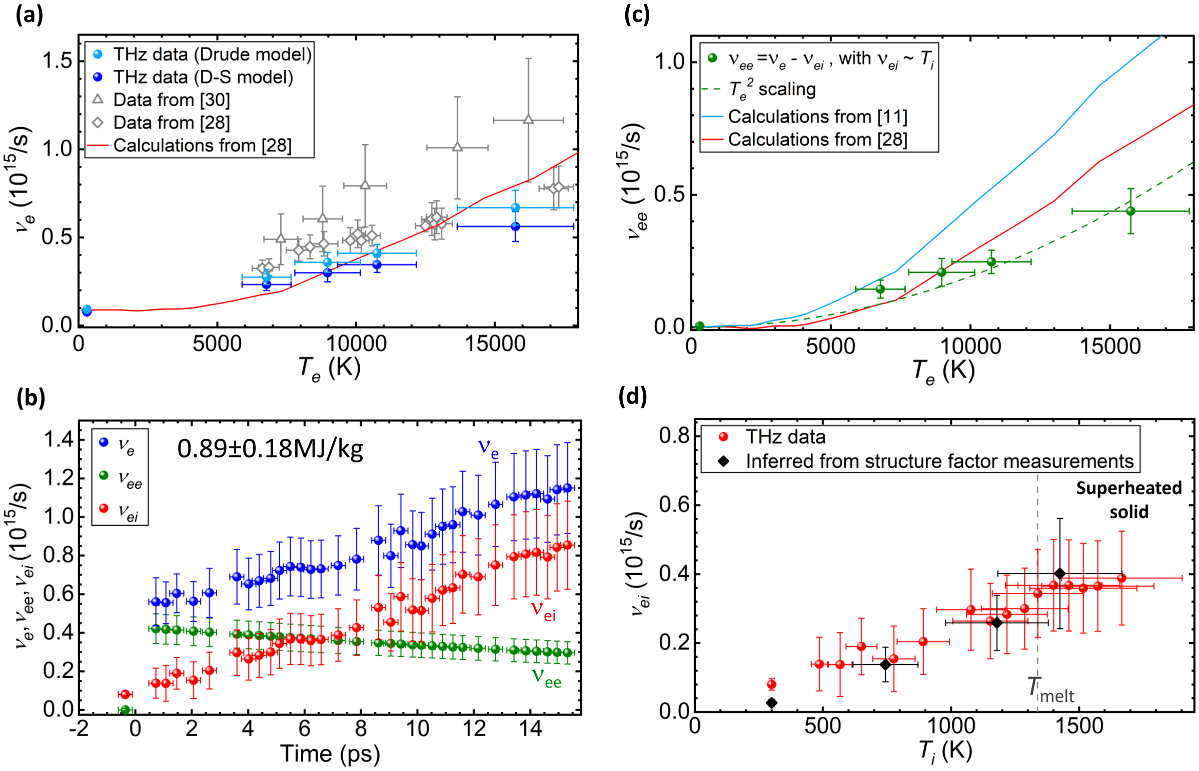

Figure S10: Electron scattering frequencies as functions of time and temperatures determined from broadband conductivity fitting by the Drude-Smith model. **(a)** shows the total electron scattering frequency (ν_e) as a function of T_e determined from this work (blue symbols), compared to the data reported from optical measurements (*28, 30*) (grey open triangles and diamonds) and the theoretical calculations from Ref. (*28*) (red curve). The ν_e data determined from the Drude model (light blue) are also show. **(b)** shows ν_e determined from the temporally resolved THz conductivity measurements (light blue symbols), which are the sum of the electron-electron scattering ν_ee (green symbols) and the electron-ion scattering frequencies ν_ei red symbols). **(c)** shows ν_ee data obtained from the total electron scattering frequency ν_e (green symbols), and the light green dashed curve is a fit to the data with a T_e^2 scaling. The blue and red curves are the theoretical calculations from Refs. (*11, 28*). **(d)** shows ν_ei data as a function of ion temperature (red symbols), and the black diamonds represent ν_ei calculated from the ion-ion structure factor (S_ii) measured in (*12*) utilizing the Ziman formula (*32*), indicating excellent agreement between these two methods. The horizontal error bars in (a), (c) and (d) taken into account the FEL energy fluctuation and temperature distribution over the THz probed area, and in (b) they represent the width of the THz cycles. The vertical error bars are determined by the precision of the measured electrical conductivity and the error bars of the electron temperature takes into account both the uncertainties of the experimental measurements and the range of temperatures as determined by the TTM calculations.

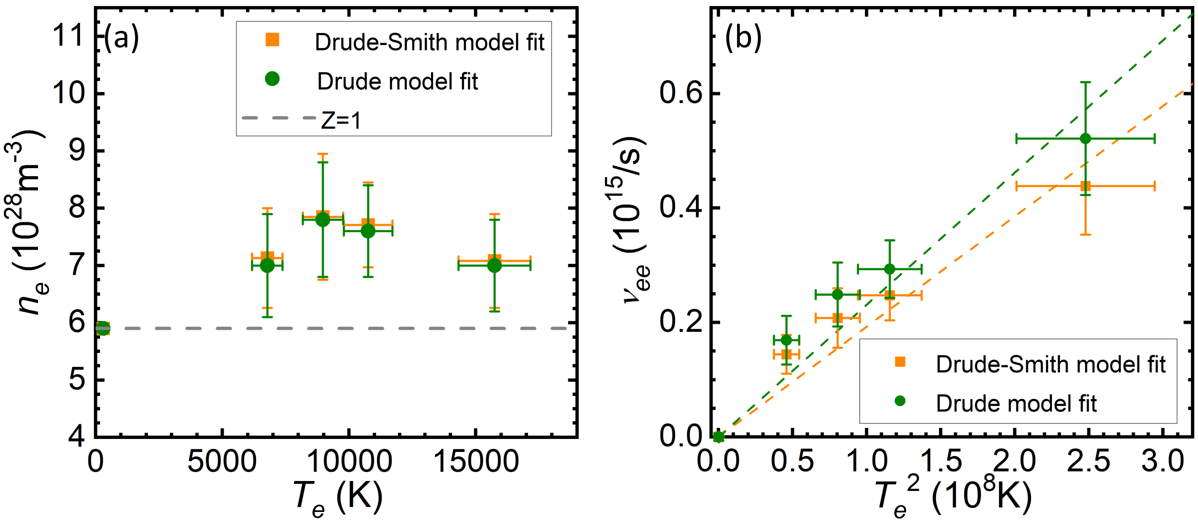

Figure S11: The comparison of parameters (a) n_e and (b) ν_ee that obtained from the Drude-Smith model (orange squares) and Drude model (green circles) by fitting the broadband conductivity σ_r at different electron temperature conditions measured at time delay t=0.7±0.2 ps. The data points from low to high temperature values correspond to absorbed energy densities of 0, 0.13±0.03, 0.24±0.05, 0.36±0.07 and 0.9±0.18 MJ/kg, respectively. The error bars are inherent from the experimental measurements of σ_r.

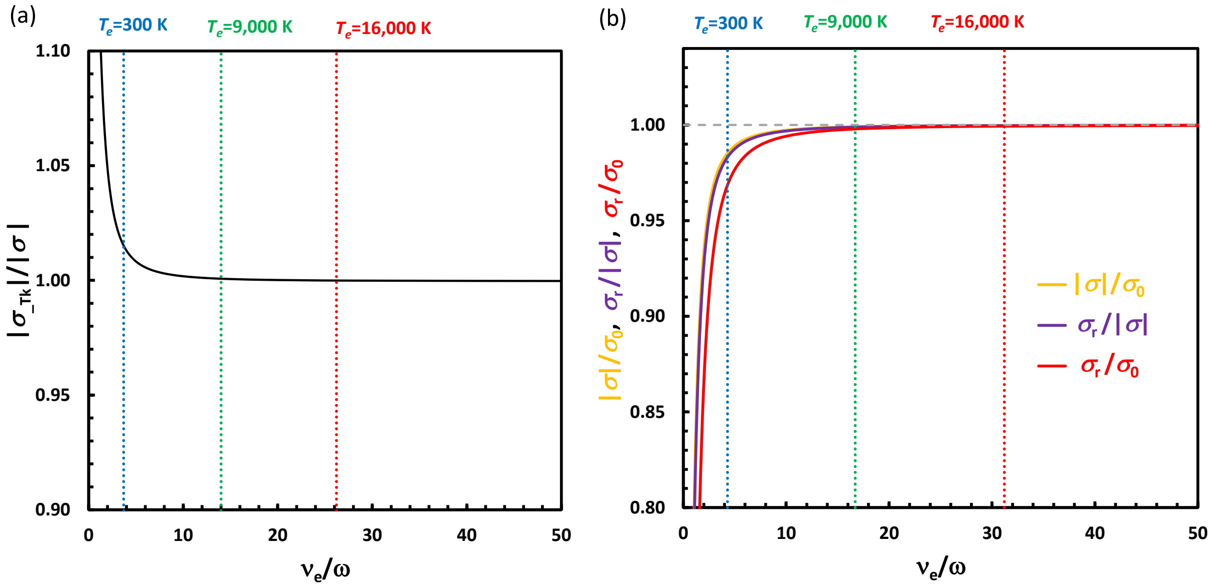

Figure S12: (a) The ratio of $|\sigma_{Tk}|$ calculated from the Tinkham equation over the exact value of $|\sigma|$ is shown as a function of electron momentum scattering frequency $\nu_{\rm e}$ over the probe electro-magnetic field frequency ω, with $\omega/(2\pi)$ = 2.85THz. (b) The ratios of $|\sigma|/\sigma_0$, $\sigma_r/|\sigma|$ and σ_r/σ_0 are shown as functions of $\nu_{\rm e}/\omega$. Here σ, σ_r and σ_0 are synthetic data obtained from the Drude-Smith model with the ratios of $\nu_{\rm e}/\omega$ that cover the range of our measurements, e.g.. the $\nu_{\rm e}/\omega$ ratios for different excitation conditions that produces peak $T_{\rm e}$ of 300 K, 9000 K and 16000 K are marked by dotted lines.

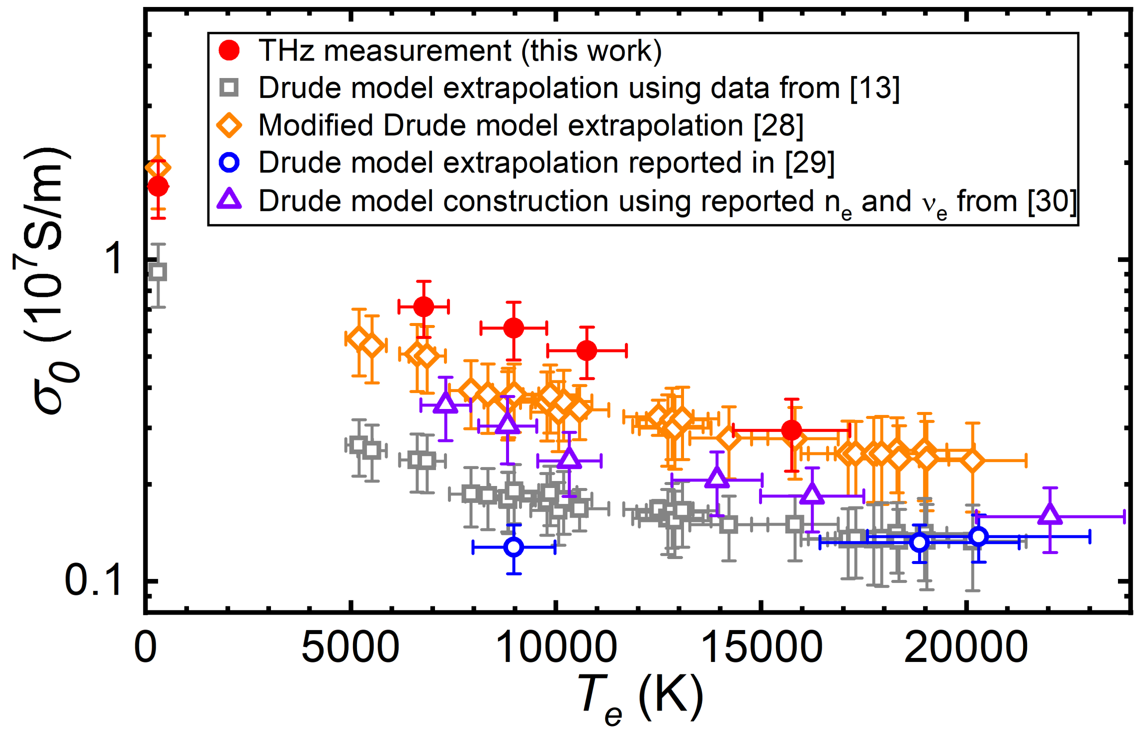

Figure S13: Comparison of the DC conductivity (σ_0) of isochorically heated gold as a function of electron temperature (T_e) determined from our THz transmission measurements (red solid circles), and the extrapolation results from the optical conductivity of (*13*) (grey open squares) and (*29*) (open blue circles) using the original Drude model and a modified Drude model (*28*) (orange open diamonds). Also shown is the DC conductivity constructed by the Drude model using electron density n_e and scattering frequency ν_e reported in (*30*) (purple open triangles). The error bars in THz represents the systematic uncertainties and standard deviations of our measurements.

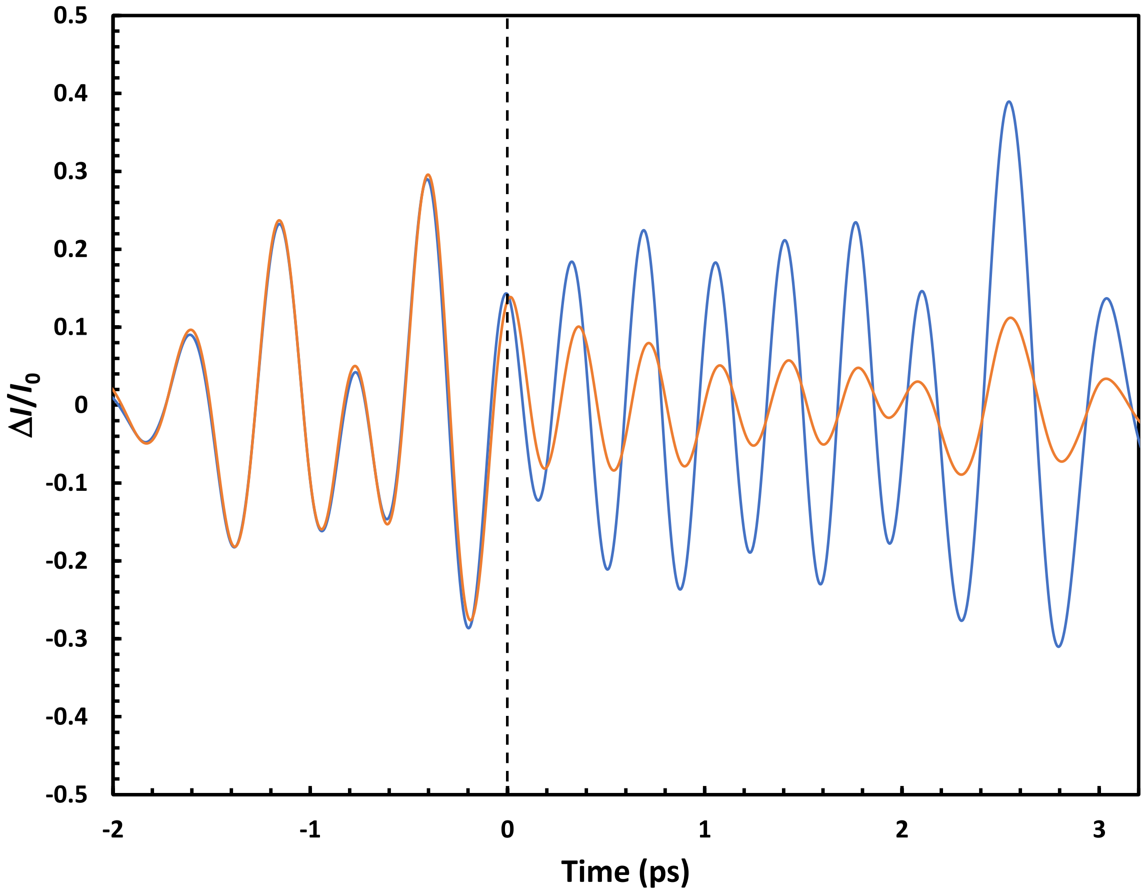

Figure S14: THz electric-field transmission through 500μm-thick silicon wafer without XUV-FEL excitation (blue curve), and with XUV-FEL excitation when the XUV pulse arrives at 0 ps (orange curve).

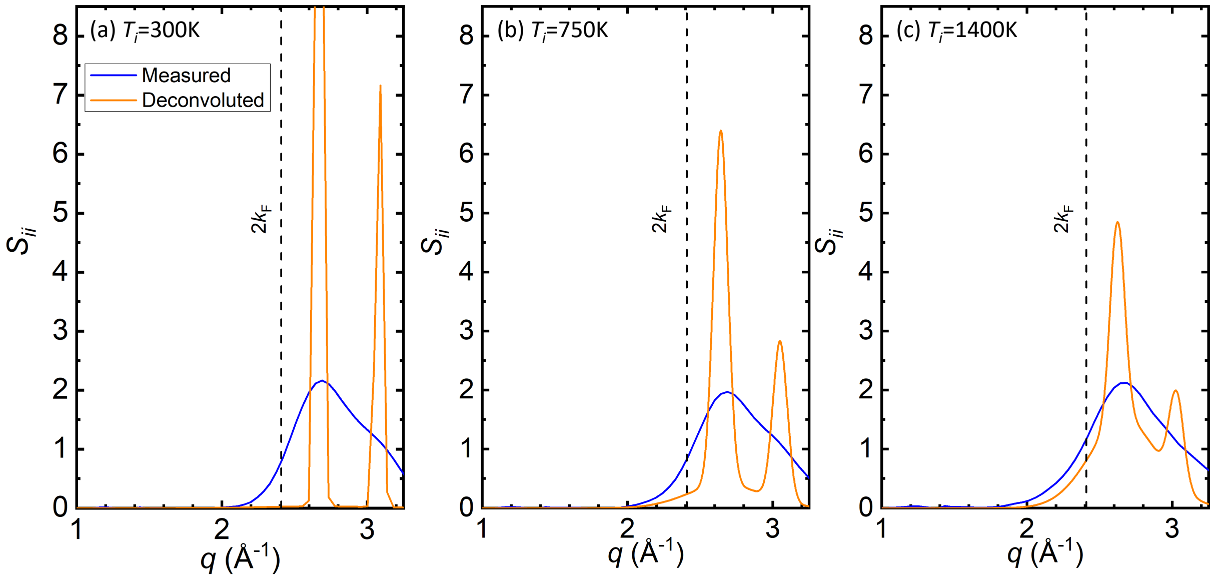

Figure S15: Ion-ion structure factor ($S_{\rm ii}$) of ultrafast laser heated gold as a function of the diffraction vector q is shown for ion temperatures of (a) $T_{\rm i}$= 300 K (room temperature solid), (b) 750 K (hot solid) and (c) 1400 K (super-heated solid) measured by ultrafast electron diffraction (*12*). The blue curves are the data from the original measurement, and the orange curves are the results deconvoluted to account for the instrumental resolution. The vertical dashed lines mark twice the Fermi wave vector $2k_{\rm F} = 2p_{\rm F}/\hbar$.

References

1. Henke, B., Gullikson, E. & Davis, J. X-ray interactions: photoabsorption, scattering, transmission, and reflection at e=50-30000 ev, z=1-92. *At. Data Nucl. Data Tables* **54**, 181 (1993).

2. Yeh, J. J. & Lindau, I. Atomic subshell photoionization cross sections and asymmetry parameters: $1 \leq z \leq 10^3$. *At. Data Nucl. Data Tables* **32**, 1 (1985).

3. Medvedev, N., Zastrau, U., E.Forster, Gericke, D. & Rethfeld, B. Short-time electron dynamics in aluminum excited by femtosecond extreme ultraviolet radiation. *Phys. Rev. Lett.* **107**, 165003 (2011).

4. Geloni, G. *et al.* Coherence properties of the european xfel. *New J. Phys.* **12**, 035021 (2010).

5. Nagler, B., Zastrau, U., Fäustlin, R. R. & $etal.$. Turning solid aluminium transparent by intense soft x-ray photoionization. *Nat. Phys.* **5**, 693 (2009).

6. Chen, Z., Sametoglu, V., Tsui, Y. Y., Ao, T. & Ng, A. Flux-limited nonequilibrium electron energy transport in warm dense gold. *Phys. Rev. Lett.* **108**, 165001 (2012).

7. Chen, Z. *et al.* Interatomic potential in the nonequilibrium warm dense matter regime. *Phys. Rev. Lett.* **121**, 075002 (2018).

8. Anisimov, S., Kapeliovich, B. & Perel'man, T. Electron emission from metal surfaces exposed to ultrashort laser pulses. *Sov. Phys.-JETP* **39**, 375 (1974).

9. Holst, B. *et al.* Ab initio model of optical properties of two-temperature warm dense matter. *Phys. Rev. B* **90**, 035121 (2014).

10. Cordoba, G. & Brooks, C. R. The heat capacity of gold from 300 to 1200 k: Experimental data and analysis of contributions. *Phys. Stat. Sol. (a)* **6**, 581 (1971).

11. Petrov, Y. V., Inogamov, N. & Migdal, K. Thermal conductivity and the electron–ion heat transfer coefficient in condensed media with a strongly excited electron subsystem. *JETP Lett.* **97**, 20 (2013).

12. Mo, M. Z. *et al.* Heterogeneous to homogeneous melting transition visualized with ultrafast electron diffraction. *Science* **360**, 1451 (2018).

13. Chen, Z. *et al.* Evolution of ac conductivity in nonequilibrium warm dense gold. *Phys. Rev. Lett.* **110**, 135001 (2013).

14. Tiedtke, K. *et al.* The soft x-ray free-electron laser flash at desy: beamlines, diagnostics and end-stations. *New J. Phys* **11**, 023029 (2009).

15. Groeneveld, R. H. M., Sprik, R. & Lagendijk, A. Femtosecond spectroscopy of electron-electron and electron-phonon energy relaxation in ag and au. *Phys. Rev. B* **51**, 11433 (1995).

16. Kruglyak, V. *et al.* Measurement of hot electron momentum relaxation times in metals by femtosecond ellipsometry. *Phys. Rev. B* **71**, 233104 (2005).

17. Teo, S. M., Ofori-Okai, B. K., Werley, A. & Nelson, K. A. Single-shot thz detection techniques optimized for multidimensional thz spectroscopy. *Rev. Sci. Instr.* **86**, 051301 (2015).

18. Tinkham, M. Energy gap interpretation of experiments on infrared transmission through superconducting films. *Phys. Rev.* **104**, 845 (1956).

19. Gensch, M. *et al.* New infrared undulator beamline at flash. *Infrared Phys. Technol.* **51**, 423 (2008).

20. Tavella, F., Stojanovic, N., Geloni, G. & Gensch, M. Few-femtosecond timing at fourth-generation x-ray light sources. *Nat. Photon.* **5**, 162 (2011).

21. Mo, M. *et al.* Visualization of ultrafast melting initiated from radiation-driven defects in solids. *Sci Adv.* **5**, eaaw0392 (2019).

22. Walther, M. *et al.* Terahertz conductivity of thin gold films at the metal-insulator percolation transition. *Phys. Rev. B* **76**, 125408 (2007).

23. Smith, N. V. Classical generalization of the drude formula for the optical conductivity. *Phys. Rev. B* **64**, 155106 (2001).

24. Ashcroft, N. W. & Mermin, N. D. *Solid State Physics* (Harcourt Brace Jovanovich Publishers, Toronto, 1976).

25. Johnson, P. B. & Christy, R. W. Optical constants of the noble metals. *Phys. Rev. B* **6**, 4370 (1972).

26. Olmon, R. L. *et al.* Optical dielectric function of gold. *Phys. Rev. B* **86**, 235147 (2012).

27. Born, M. & Wolf, E. *Principles of Optics, 6th ed.,* (Pergamon Press, Oxford, 1980).

28. Ng, A., Sterne, P., Hansen, S. & Recoules, V. t. dc conductivity of two-temperature warm dense gold. *Phys. Rev. E* **94**, 033213 (2016).

29. Widmann, K. & *et al.* Single-state measurement of electrical conductivity of warm dense gold. *Phys. Rev. Lett.* **92**, 125002 (2004).

30. Fourment, C. *et al.* Experimental determination of temperature-dependent electron-electron collision frequency in isochorically heated warm dense gold. *Phys. Rev. B* **89**, 116110(R) (2014).

31. Cooke, D. G. *et al.* Transient terahertz conductivity in photoexcited silicon nanocrystal films. *Phys. Rev. B* **73**, 193311 (2006).

32. Ziman, J. M. A theory of the electrical properties of liquid metals. i: The monovalent metals. *Philos. Mag.* **6**, 1013 (1961).

33. Evans, R., Gyorffy, B. L., Szabo, N. & Ziman, J. M. *The Properties of Liquid Metals, edited by S. Takeuchi* (Wiley, New York, 1973).

34. Reinholz, H., Redmer, R., Ropke, G. & Wierling, A. Long-wavelength limit of the dynamical local-field factor and dynamical conductivity of a two-component plasma. *Phys. Rev. E* **62**, 5648 (2000).

35. Reinholz, H. Dielectric and optical properties of dense plasmas. *Ann. Phys. (Paris)* **30 (4)**, 1 (2005).

36. Kremp, D., Schlanges, M. & Kraeft, W.-D. *Quantum Statistics of Nonideal Plasmas* (Springer, Berlin, Heidelberg, 2005).

37. Gregori, G., Glenzer, S. H., Rozmus, W., Lee, R. W. & Landen, O. L. Theoretical model of x-ray scattering as a dense matter probe. *Phys. Rev. E* **67**, 026412 (2003).

38. Doyle, P. & Turner, P. Relativistic hartree-fock x-ray and electron scattering factors. *Acta Cryst.* **A24**, 390 (1968).

39. Lin, Z. & Zhigilei, L. V. Time-resolved diffraction profiles and atomic dynamics in short-pulse laser-induced structural transformations: Molecular dynamics study. *Phys. Rev. B* **73**, 184113 (2006).
